# Prognostic indicators of arthroscopic discopexy for management of temporomandibular joint closed lock

**DOI:** 10.1038/s41598-022-07014-9

**Published:** 2022-02-24

**Authors:** Manoj Kumar Sah, Ahmed Abdelrehem, Shihui Chen, Pei Shen, ZiXian Jiao, Ying Kai Hu, Xin Nie, Chi Yang

**Affiliations:** 1 Department of Oral Surgery, Shanghai Ninth People’s Hospital, Shanghai Jiao Tong University School of Medicine, 639 Zhizaoju Road, Shanghai, 200011 China; 2 College of Stomatology, Shanghai Jiao Tong University School of Medicine, Shanghai, China; 3 Shanghai Key Laboratory of Stomatology & Shanghai Research Institute of Stomatology, National Clinical Research Center of Stomatology, Shanghai, China; 4 Department of Craniomaxillofacial and Plastic Surgery, Faculty of Dentistry, Alexandria University, Alexandria, Egypt; 5 Biostatistics Office of Clinical Research Unit, Shanghai Ninth People’s Hospital, Shanghai Jiao Tong University School of Medicine, Shanghai, China

**Keywords:** Dental diseases, Oral diseases

## Abstract

In order to optimize patient selection for temporomandibular joint (TMJ) arthroscopic discopexy to achieve favorable outcomes, prognostic indicators impacting the results are important to analyze. This longitudinal retrospective study aimed to analyze various prognostic factors impacting surgical outcomes following arthroscopic discopexy for management of TMJ closed lock using success criteria based on pain, maximal interincisal opening, diet, and quality of life. Furthermore, a quantitative MRI assessment was performed pre- and post-operatively. Multivariate analysis was used to evaluate various prognostic variables including gender, age, side, duration of illness, Wilkes staging, parafunctional habits, splint therapy and orthodontic treatment. A total of 147 patients (201 joints) were included. The outcome was categorized as excellent (n = 154/76.61%), good (n = 34/16.91%), or poor (n = 13/6.46%) with a success rate of 93.54%. Patients aged > 30 years old (*p* = 0.048), longer duration of illness (12–24 months: *p* = 0.034) and (> 24 months: *p* = 0.022), and patients with Wilkes stage IV (*p* = 0.002) were all significantly more likely to be in the poor outcome group. Finally, orthodontic treatment showed a significant association with excellent outcomes (*p* = 0.015). Age, duration of illness, Wilkes staging, and orthodontic treatment are considered significant prognostic factors that can predict the outcomes following the arthroscopic discopexy for management of TMJ closed lock.

## Introduction

Anterior disc displacement (ADD) of the temporomandibular joint (TMJ) is so far the most frequently encountered form of TMJ internal derangement (ID) with an estimated incidence rate of 36% of the general population, of which 81.25% was found to be pure ADD, more prevailing in females at their twenties^1^. The clinical scenario of ADD commonly consists of pain, joint sounds, limitation of mouth opening, closed lock and might be associated with osteoarthritic changes at late disease stages^2,3^. Most patients get benefit of the conservative treatments that include medical therapies, physiotherapy and occlusal splints. However, still a considerable proportion of patients with a predominance of degenerative findings is refractory for such regimen after a period of non-interventional treatment, for whom, surgical procedures are being advocated including disc repositioning techniques^4,5^.

Among various surgical interventions for management of TMJ closed lock, the arthroscopic disc suturing and repositioning technique is now gaining more popularity than the conventional open approaches due to development of new technologies and refinement of the provisional methods^6–8^. Reviewing the literature, several scholars have tried to reposition the disc arthroscopically with different suturing techniques, but the success rate and long-term stability have not been satisfactory^9–11^. Since the original reports of arthroscopic disc suturing^12–14^, such technique has passed through different approaches with various rates of success and outcomes. Then, McCain et al.^9^ reported an eminent technique of arthroscopic disc repositioning and suturing, with a success rate of 81.8%, however with smaller sample size (11 joints). Later, Yang et al.^15^ introduced a new technique of arthroscopic TMJ disc repositioning and suturing, applied on 2167 subjects (2622 joints), with a higher success rate (98.07%), demonstrated in the short- and long-term follow ups evidenced in magnetic resonance imaging (MRI)^16^.

The postoperative outcomes of TMJ arthroscopy have been analyzed in previous literature reports based only on individual clinical factors^17,18^ while, very specific studies related the disc repositioning procedure to both clinical and MRI data^19^. However, they all lack the followings: (1) most reports analyzed a single variable, and seldom reported combined variable effects; (2) all previous articles analyzed the outcomes of arthroscopic lysis and lavage with paucity of reports investigating the arthroscopic disc repositioning procedure correlated with different variables.

Notwithstanding the higher success rate reported with Yang’s arthroscopic technique^15,16^, still some cases have been reported with unfavorable outcomes at different follow up intervals. Therefore, the authors suppose that a multivariate regression analysis of different variables impacting the outcomes, would be critical in terms of prognosis and better patient selection. The current study aimed to retrospectively analyze different prognostic factors including gender, age, side, duration of illness, Wilkes staging, parafunctional habits, splint therapy and orthodontic treatment to correlate with the clinical and radiological outcomes of TMJ arthroscopic disc repositioning in a multivariate regression analysis model.

Thus, in the current study, the null hypothesis assumed that there will be no significant differences between different prognostic factors affecting the surgical outcomes following an arthroscopic disc suturing and repositioning technique for management of TMJ closed lock.

## Methods

### Study design

The current research retrospectively included all consecutive patients diagnosed as closed lock and were operated with Yang’s arthroscopic disc repositioning and suturing technique from March, 2014 to September, 2016 in the Department of Oral Surgery, Shanghai Ninth People’s Hospital, Shanghai Jiao Tong University, School of Medicine. This study was accomplished in accordance with the principles outlined in the Declaration of Helsinki, with an approval of the Ethics Committee of Shanghai Ninth People’s Hospital (Ethical Vote Number: SH9H-2020-T34-2, date of approval: April 29th, 2020). An informed written agreement was obtained from all participants.

All arthroscopic procedures were performed by one senior surgeon (C.Y.), with more than 40 years’ experience.

### Inclusion criteria

1. Patients having at least one recent preoperative MRI (within 3 months);
2. Having a minimum of two postoperative MRI scans (at least 6 months apart);
3. Patients presenting with stages III and IV (Wilkes classification)^20^;
4. Patients operated with Yang’s TMJ arthroscopic disc suturing and repositioning technique;
5. Adequate pre and postoperative clinical data; compliant patients who were followed up in the proposed intervals with clinical measurements.

### Exclusion criteria

Patients were excluded as study subjects if:

1. Patients with septic arthritis or synovial chondromatosis;
2. Psychological disorders;
3. Joints operated before for any other TMJ problems;
4. Insufficient or unclear MRI data;
5. Missing clinical data or follow ups of patients.

### Study variables

*The primary predictor variables* comprised different prognostic variables including gender, age, side, duration of illness, Wilkes staging, parafunctional habits, splint therapy and orthodontic treatment. *The primary outcome variables* were composed mainly of the clinical outcomes [maximal interincisal opening (MIO), pain, diet, and quality of life (QOL)]. *The secondary outcome variables* consisted of the MRI-based radiological outcomes (disc position and condylar height).

### Arthroscopic procedure^15^

Local anesthesia was applied in all arthroscopic surgeries that consisted mainly of four portals of entry. Following fossa portal entry, a “2.3 mm arthroscope system” (Stryker, San Jose, CA, USA) with a “2.8 mm outer protective cannula”, was introduced into the posterior recess, followed by thorough examination of the joint cavity to confirm the diagnosis of ADD. Under a complete arthroscopic visualization, the eminence portal was achieved, through which the “coblation probe” (ArthroCare System 2000; ArthroCare, Sunnyvale, CA, USA) accessed the anterior recess for anterior release. Through a midway access point between the first two punctures, a “12-gauge needle” was introduced into the upper joint space to penetrate the TMJ disc 1–2 mm ahead of the junction between the body of the cartilaginous disc and posterior retrodiscal tissue. A third transmeatal puncture was made, through which the “custom-made suture needles” (Shanghai ShenDing Industrial, Shanghai, China) were inserted. A “specially-manufactured non-absorbable surgical suture material” (Shanghai Pudong Golden Ring, Shanghai, China) was passed from the “12-gauge needle” and pulled out via the transmeatal portal with the help of the “lasso- and hook-type grippers”. Similarly, a second suture was made for better securing the disc position. A “stabilizing splint” was used for all patients after surgery. Such splints were constructed based on the jaw with better teeth alignment either maxilla or mandible. A master cast was fabricated by taking an “alginate impression” of the arch. To construct a soft stabilizing splint, a “vacuum pressure molding device” was used for fabrication with “2-mm thick rubber sheets” measuring 13 × 13 cm.

### Evaluation and workflow analysis of prognostic variables (Fig. 1)

**Figure 1:**
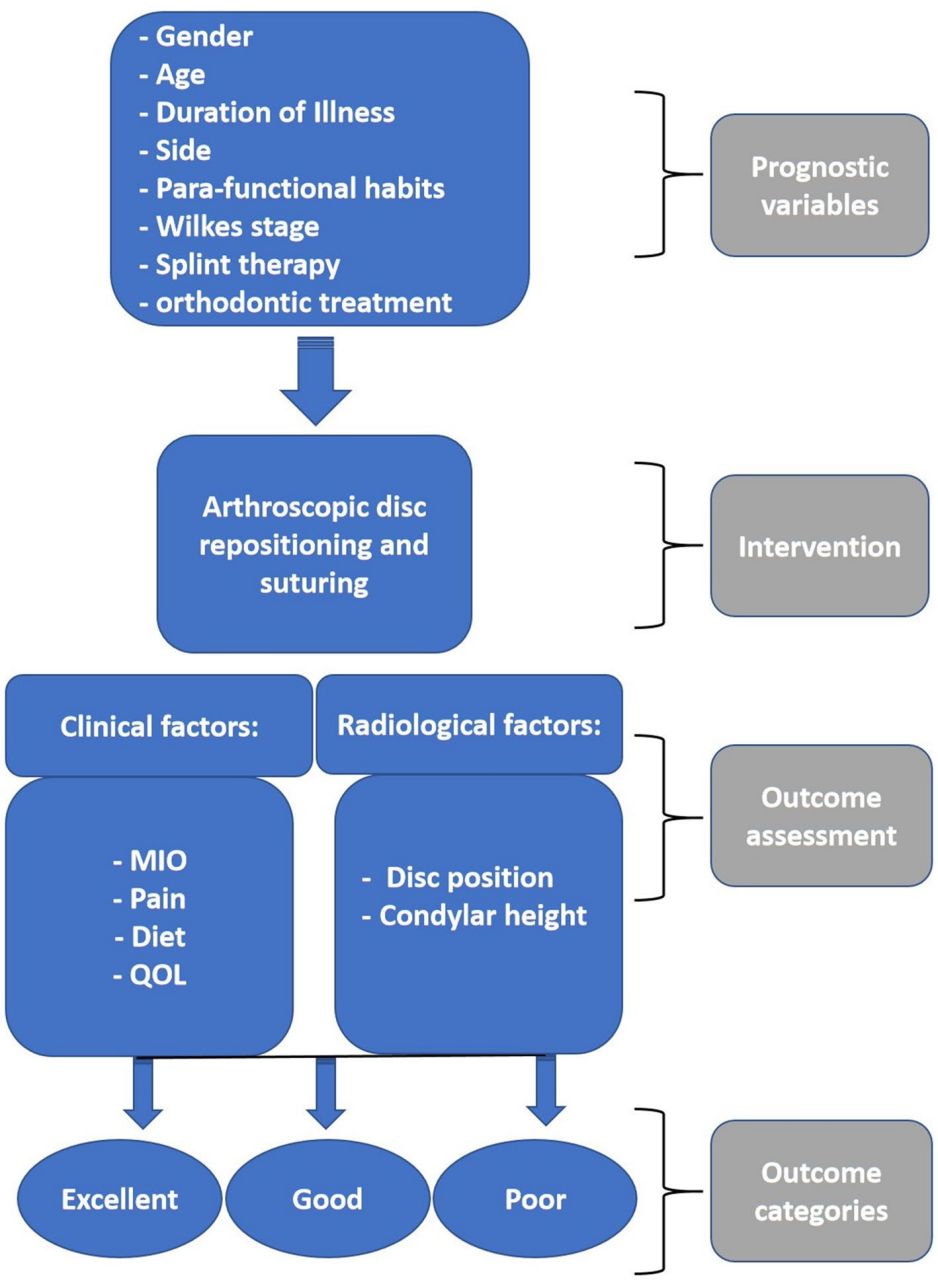
Study design and workflow analysis of prognostic variables.

All patients’ records including gender, age, side, duration of illness, Wilkes staging, parafunctional habits, splint therapy, and orthodontic treatment were reviewed and analyzed. Also, MRI data were reviewed for disc position and condylar height.

All preoperative patient data were input into an ordered multivariate analysis to calculate a prognostic model for success or failure following the arthroscopic disc repositioning operations. The outcome items included:

1. Maximal interincisal opening (MIO), (success ≥ 35 mm);
2. Pain scores measured with visual analogue scale (VAS), (success ≤ 3);
3. Diet scores (success ≤ 3);
4. Quality of life (QOL) (4 was indicative of success).

The definitions and ratings of the outcome measures are all summarized in Table 1.

**Table 1:** Definitions and ratings of different outcome measures.

Outcome criteria	Definition	Rating
MIO	Objective measurement using an ordinary ruler with both jaws at greatest opening without help (estimating distance between the lower and upper incisor tips)	≥ 35 mm mouth opening is categorized as successful
Pain	Subjective rating using a numeric visual analogue scale (VAS) ranging from 0 to 10, where 0 = no pain 10 = worst pain imaginable	VAS ≤ 3 is regarded successful
Diet	Subjective rating using a numeric visual analogue scale (VAS) ranging from 1 to 10, where 1 = regular diet 10 = only liquid	Success ≤ 3
QOL	Subjective rating using a numeric visual analogue scale (VAS) ranging from 1 to 4 1 = rest in bed 4 = ordinary daily life activities	4 is indicative of success

The outcomes of arthroscopic disc repositioning and suturing were categorized into three different subgroups namely; excellent, good, and poor based on the analyzed postoperative data for more than 4 years.

An excellent outcome was confirmed when all 4 clinical parameters were fulfilled (MIO ≥ 35 mm, pain ≤ 3, diet ≤ 3, and QOL = 4). A good result was defined if any of 2 or 3 clinical parameters were met. A poor outcome was defined if one or none criterion was fulfilled.

### Clinical follow-up assessment^8^

MIO was measured using a conventional ruler preoperatively and at 3, 6, 12, 24, 36 and 48 months postoperatively. Pain was evaluated using a 10 cm VAS scale (where ‘0’ means no pain and ‘10’ representing the worst pain) preoperatively and at all postoperative follow-up intervals. The median VAS score at the final postoperative visit was compared with the preoperative VAS score. Diet was also evaluated using VAS scale from 1 (regular diet) to 10 (only fluids) and QOL assessmnt was done using a four point VAS scale from 1 (rest in bed) to 4 (ordinary daily activities) at pre- and postoperative visits.

### MRI acquisition and evaluation

MRI scans were obtained from a 1.5-Tesla imager (Signa; General Electric, Milwaukee, WI, USA) with 3-inch TMJ surface coil receivers on bilateral sides. The parasagittal eight sections of T1-weighted spin-echo sequence scan from lateral to medial for each TMJ in the closed mouth position were first examined using a computer dataset (Y410P; Lenovo Computer, Beijing, China). The central image displaying the largest sectional area (usually the fourth or fifth slice) of the condyle was selected for tracing the reference planes and drawing the layouts of the joint structures by utilizing Adobe Photoshop CS5 (Adobe Systems, San Jose, CA, USA). Besides, the linear measurements for condylar height and disc position were estimated by utilization of MB-Ruler estimating programming (Markus Bader, Berlin, Germany; precise to 0.01 mm). The construction of the tangent line at the posterior border of the ramus to assess condylar height was determined based on the method described by Markic et al.^21^ while the disc displacement distance was determined according to the method of Xie et al.^22^.

#### Assessment of condylar height

**Figure 2:**
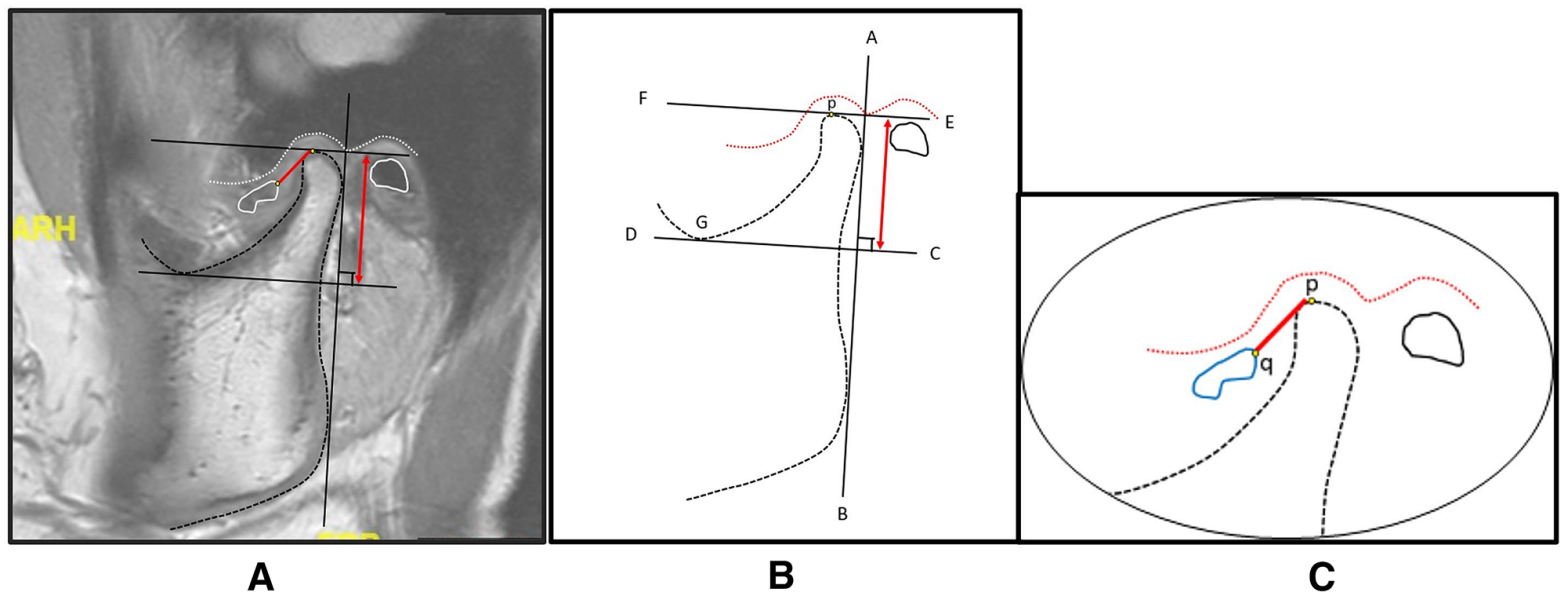
An illustrated diagram demonstrating the key reference points on MRI measurements before surgery. (**A**): A preoperative MRI sagittal view; (**B**): A schematic diagram showing reference points and lines for condylar height measurement; (**C**): A schematic diagram demonstrating reference points and lines for measurement of disc displacement distance.

**Figure 3:**
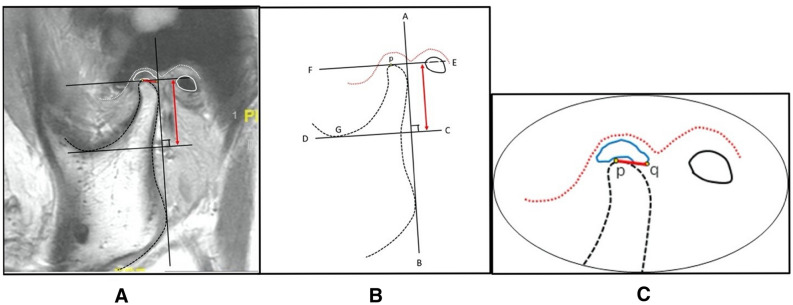
An illustrated diagram demonstrating the key reference points on MRI measurements after surgery. (**A**): A postoperative MRI sagittal view; (**B**): A schematic diagram showing reference points and lines for condylar height measurement; (**C**): A schematic diagram demonstrating reference points and lines for measurement of disc reposition distance.

For every MRI image, three points; condylion (p), disc point (q), and incisura (G) were defined, and two linear measurements were drawn perpendicular to the tangent at the posterior border of the ramus. A tangent line (AB) was drawn at the posterior border of the ramus passing through two points; first: the most cranial and convex bulge on the back of the condyle, second: the most caudal and convex bulge on the back of the ramus. From line AB, two perpendicular lines CD and EF were drawn passing through the deepest point of the sigmoid notch (G) and the top point of the condylar head (p). The distance between lines CD and EF was defined as condylar height (Figs. 2 and 3).

#### Measurement of disc position

Another point (point q) at the posterior most convex point of the disc was identified. A straight line drawn from point p to point q was represented as disc displacement/reposition distance relative to the condyle (Figs. 2 and 3).

### Statistical analysis

Analyses were carried out using a dedicated statistical software (SPSS, Version 25.0; IBM, Armonk, NY, USA). A preliminary analysis was accomplished using univariate analysis to evaluate the specific patient prognostic factors on their surgical outcomes. Chi-square test was used for binary variables including gender, side and Wilkes stages whereas Spearman regression test was applied to analyze the two ordered multiple categorical variables (age group and duration of illness). Those variables achieving a *p*-value of less than 0.1 were enrolled in the multivariate analysis. An ordered multivariate analysis was used to investigate the correlation between the prognostic variables and the surgical outcomes with an excellent outcome being set as a reference. Gender and side variables were used in an adjusted ordered multivariate model to address the possible confounders. Furthermore, Wilcoxon signed ranked test was conducted to analyze the association of disc position and condylar height differences as well as to evaluate the clinical outcomes pre- and post-operatively. Probabilities (*p*-value) of less than 0.05 were considered significant.

## Results

### Description of patients

A total of 234 joints (169 patients) were enrolled in the present study in the primary survey stage. Of these, 33 joints (22 patients) were excluded (21 joints at the borderline of either Wilkes staging II or V and 12 joints for poor MRI images), so the final total sample size was 201 joints (147 patients) being enrolled in the final analysis, including 133 females (90.47%) and 14 males (9.52%) with a mean (± SD) age of 20.26 (± 7.27) years. The right side was involved in 97 joints (48.25%) while the left side was operated in 104 joints (51.75%). The mean (± SD) value for duration of illness was 23.12 (± 18.03) months (range: 6–84 months), while the mean (± SD) value of the postoperative follow up period was 41.57 (± 6.05) months (range: 25.5–49.5 months). There were 154 joints (76.61%) in the excellent group, 34 joints (16.91%) in the good outcome group with a total success rate of 93.54%, while poor outcome was found in 13 joints (6.46%). Of the 13 failed joints, 8 joints were reoperated arthroscopically, while 5 joints were repeated with open discopexy procedure. All demographic and basic data of the included subjects are summarized in Table 2.

**Table 2:** Demographics and clinicopathologic characteristics of patients based on their surgical outcomes. Univariate analysis of the influence of patient-specific prognostic factors.

	Poor (n = 13)	Good (n = 34)	Excellent (n = 154)	Statistics (*ρ/x*^*2*^)	*p*-value
**Gender**					
Female	12 (92.3%)	31 (91.1%)	140 (90.9%)	0.03	0.985
Male	1 (7.6%)	3 (8.8%)	14 (9.0%)		
**Age group**					
< 20 Y	5 (38.4%)	17 (50.0%)	98 (63.6%)	− 0.16	0.020*
20–30 Y	7 (53.8%)	11 (32.3%)	47 (30.5%)		
> 30 Y	1 (7.6%)	6 (17.6%)	9 (5.8%)		
**Duration of illness**					
< 12 M	1 (7.6%)	17 (50.0%)	86 (55.8%)	− 0.18	0.013*
12–24 M	4 (30.7%)	8 (23.5%)	32 (20.7%)		
> 24 M	8 (61.5%)	9 (26.4%)	36 (23.3%)		
**Sides**					
Left	8 (61.5%)	14 (41.1%)	82 (53.2%)	2.16	0.34
Right	5 (38.4%)	20 (58.8%)	72 (46.7%)		
**Wilkes stage**					
III	2 (15.3%)	14 (41.1%)	99 (64.2%)	16.01	< 0.001*
IV	11 (84.6%)	20 (58.8%)	55 (35.7%)		
**Parafunctional habits**					
Yes	11 (84.6%)	24 (70.5%)	83 (53.9%)	7.05	0.029*
No	2 (15.3%)	10 (29.4%)	71 (46.1%)		
**Splint therapy**					
Yes	6 (46.1%)	13 (38.2%)	47 (30.5%)	1.87	0.393
No	7 (53.8%)	21 (61.7%)	107 (69.4%)		
**Orthodontic treatment**					
Yes	6 (46.1%)	16 (47.0%)	105 (68.1%)	7.08	0.029*
No	7 (53.8%)	18 (52.9%)	49 (31.8%)		

*x*^*2*^-Chi-square test (for binary variable including gender, side and Wilkes stage).

*ρ*-Spearman regression test (to analyze the two ordered multiple categorical variable: age group and duration of illness).

*n* number of joints, *Y* years, *M* months.

*Statistically significant.

### Clinical outcomes

As revealed by Wilcoxon signed ranked test, MIO significantly improved from preoperative measurement to the final postoperative visit [median, range: 27 mm (22–30 mm) to 40 mm (37–43 mm)], while TMJ pain VAS scores significantly decreased [median, range: 5 (4–6) to 0.0 (0.0–1.0)]. The other outcomes including diet VAS score [median, range: 4 (3.0–5.0) to 1 (1.0–1.0)] and QOL VAS score [median, range: 3 (2.0–3.0) to 4 (4.0–4.0)] were also significantly improved, (all *p* < 0.001) as demonstrated in Table 3.

**Table 3:** Clinical outcomes of TMJ arthroscopic discopexy.

Variables	Follow-up visits		
	Pre-operative	Post-operative (final visit-48 months)	*p*-value
	Median (q25;q75)		
MIO	27 (22; 30)	40 (37; 43)	< 0.001*
Pain	5 (4; 6)	0 (0; 1)	< 0.001*
Diet	4 (3; 5)	1 (1; 1)	< 0.001*
QOL	3 (2; 3)	4 (4; 4)	< 0.001*

*p < 0.001 (Wilcoxon signed ranked test).

### Radiological outcomes

**Figure 4:**
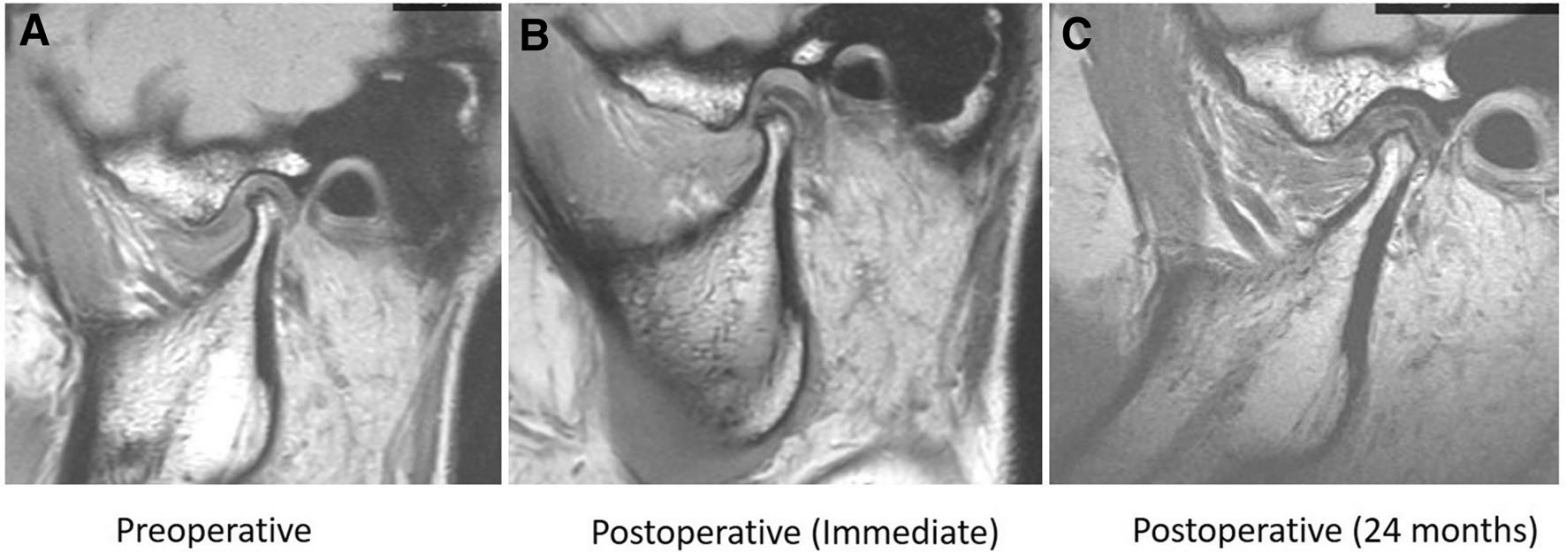
MRI scans of a 17-year-old female patient with ADD on the left side. (**A**) Preoperative MRI image, (**B**) Immediate postoperative MRI image showing successful disc repositioning, (**C**) Stable disc position with condylar bone remodeling at 24 months of postoperative follow-up.

Wilcoxon signed ranked test showed a statistically significant difference in the median quantitative measurement of condylar disc position before and immediately after surgery (*p* < 0.001), indicating successful disc repositioning. Furthermore, the disc position between postoperative initial and final visits showed significant differences (*p* < 0.001), however, the difference was minimal and this was expected to move slightly forward from its immediately postoperative overcorrected position. Meanwhile, the condylar height differences before and immediately after surgery as well as initial and final visits following surgery were all statistically significant (*p* < 0.001) (Table 4). Successful and stable disc repositioning was revealed in 188 joints (93.54%) evidenced on MRI examination at various follow-up visits (Table 4 and Fig. 4).

**Table 4:**
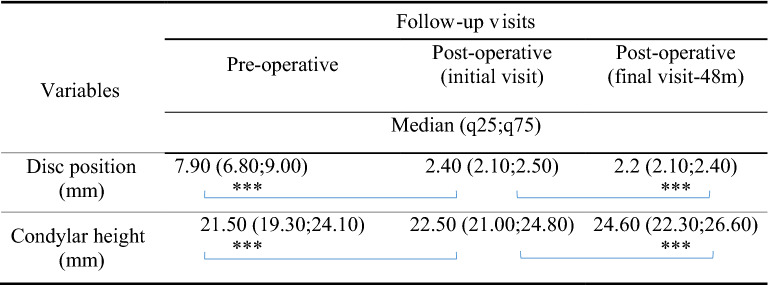
Changes in disc position and condylar height pre- and postoperatively.

***p < 0.001 (Wilcoxon signed ranked test).

### Prognostic variables and surgical outcomes

Among the individual patient related prognostic factors examined, age group, duration of illness, Wilkes stage, parafunctional habits and orthodontic therapy exhibited a statistically significant association (*p* = 0.020, *p* = 0.013, *p* < 0.001, *p* = 0.029, *p* = 0.029, respectively) with surgical outcomes. On the contrary, gender, side and splint therapy did not seem to be associated with postoperative outcomes (*p* = 0.985, *p* = 0.034, *p* = 0.393, respectively) (Table 2).

When the ordered multivariate analysis was applied in an unadjusted model, it revealed that patients aged > 30 years old (OR 0.33, 95% CI 0.11–0.99, *p* = 0.048) were significantly more likely to be in the poor outcome group compared to other age groups. Also, patients with longer duration of illness (12–24 months: OR 0.36, 95% CI 0.14–0.93, *p* = 0.034) and (> 24 months: OR 0.37, 95% CI 0.16–0.87, *p* = 0.022) were more likely to be in the poor outcome group compared to patients with < 12 months duration of illness. Furthermore, patients with Wilkes stage IV (OR 0.28, 95% CI 0.13–0.64, *p* = 0.002) were significantly associated with poor outcomes compared to Wilkes stage III. Finally, patients having an orthodontic treatment showed a positive association with excellent outcomes (OR 3.12, 95% CI 1.25–7.80, *p* = 0.015). Parafunctional habits (*p* = 0.576) and splint therapy (*p* = 0.539) were found to be insignificant variables for the surgical outcomes (Table 5).

**Table 5:** Ordered multivariate analysis (unadjusted) with prognostic variables and surgical outcomes of the TMJ arthroscopic discopexy.

	OR	95%CI	*p*-value
**Age group**			
< 20 Y	1		
20–30 Y	0.67	0.30–1.50	0.334
> 30 Y	0.33	0.11–0.99	0.048*
**Duration of illness**			
< 12 M	1		
12–24 M	0.36	0.14–0.93	0.034*
> 24 M	0.37	0.16–0.87	0.022*
**Wilkes stage**			
IV (Vs. III)	0.28	0.13–0.64	0.002*
**Parafunctional habits**			
Yes (Vs. No)	0.76	0.32–1.89	0.576
**Splint therapy**			
Yes (Vs. No)	1.34	0.53–3.38	0.539
**Orthodontic treatment**			
Yes (Vs. No)	3.12	1.25–7.80	0.015*

The parallel assumption hold (*p* = 0.100 > 0.05).

*OR* Odds ratio, *CI* Confidence interval, *Y* years, *M* months.

*Statistically significant.

Similarly, when adjusting the ordered multivariate analysis for gender and side, the durations of illness (12–24 months: OR 0.33, 95% CI 0.12–0.88, *p* = 0.026) and (> 24 months: OR 0.35, 95% CI 0.15–0.83, *p* = 0.017) were still likely to be in the poor outcome group. Also, patients with Wilkes stage IV (OR 0.27, 95% CI 0.12–0.62, *p* = 0.002) were significantly associated with poor outcomes. However, the patients having an orthodontic treatment still showed positive association with excellent outcomes (OR 3.08, 95% CI 1.24–7.70, *p* = 0.016) (Table 6).

**Table 6:** Ordered multivariate (adjusted) with prognostic variables and surgical outcomes of the TMJ arthroscopic discopexy.

	OR	95%CI	*p*-value
**Age group**			
< 20 Y	1		
20–30 Y	0.71	0.31–1.61	0.412
> 30 Y	0.36	0.12–1.11	0.075
**Duration of illness**			
< 12 M	1		
12–24 M	0.33	0.12–0.88	0.026*
> 24 M	0.35	0.15–0.83	0.017*
**Wilkes stage**			
IV (Vs. III)	0.27	0.12–0.62	0.002*
**Parafunctional habits**			
Yes (Vs. No)	0.76	0.31–1.87	0.553
**Splint therapy**			
Yes (Vs. No)	1.34	0.53–3.34	0.534
**Orthodontic treatment**			
Yes (Vs. No)	3.08	1.24–7.70	0.016*

The parallel assumption hold (*p* = 0.191 > 0.05).

*OR* Odds ratio, *CI* Confidence interval, *Y* years, *M* months.

*Statistically significant.

## Discussion

Since the original introduction of Yang’s arthroscopic disc repositioning technique, this has long been evaluated in previous reports revealing various success rates that range from 98.07 to 98.56%^8,15^. In order to optimize the outcomes of such treatment modality, different prognostic variables impacting the results are important to analyze. The selection of patients who would most likely benefit from a specified treatment option can be improved by having distinct research-based diagnostic criteria and strict selection principles. In addition, a correct identification of radiological and patient-based specific factors are important to guide the surgeons to predict treatment outcomes and to suggest the patient an overview regarding the benefits of the proposed treatment, which can prevent an unnecessary treatment for those who would not benefit. To meaningfully obtain this, individualized predictors of treatment outcomes are a prerequisite^23^. In the current study, the results confirmed the authors’ hypothesis, revealing significant differences between various prognostic factors affecting the surgical outcomes following an arthroscopic disc suturing and repositioning technique for management of TMJ closed lock, and confirming that age, duration of illness, and Wilkes staging were the most significant predictors for successful surgical outcomes.

From the results of the current study, a total success rate of 93.54% was revealed in our series, which was comparable with our previous reports^8,15^. The study confirmed that there were significant differences between age, duration of illness, Wilkes staging and associated preoperative orthodontic treatment that affect the clinical and radiological outcomes following TMJ arthroscopic disc repositioning and suturing.

Our results showed no significant differences in the surgical outcomes in correlation with the gender variable. It cannot be excluded that the current study is under-powered in terms of this specific parameter due to the low number of male patients included. A large scale study is therefore required to fully explore the question of outcome in relation to gender.

Considering the age variable, the multivariate regression analysis revealed that younger age patients were significantly associated with excellent outcomes following the arthroscopic surgery, which was consistent with the results in our previous study^8^. However, such results were not in line with the reports of previous studies^24,25^ which concluded that older age subjects were the most benefiting from an arthroscopic surgery in advanced disease stages, however, with only analysis of the outcomes following the operative arthroscopy without specifying disc suturing. Contrary, a retrospective cohort analyzing the age as the primary predictor variable, revealed that age showed no impact on the outcomes following arthroscopic surgery^17^^.^ This can be related to the large difference in the mean age of patients in their study and the current report, with more satisfactory outcomes following arthroscopy with the lower age group. In our study, age showed a significant impact on the outcomes which can be related to the fact that the retrodiscal tissues and adjacent articular structures are soft and resilient in younger age patients making the anterior release, disc recapture and suturing procedures more easier to handle than in elderly patients with more stiff tissues.

In the current report, the authors used specific patient variables as an indicator of the surgical outcomes with the design of success criteria based on MIO, pain, diet and QOL. With this multi-variable analysis, the present study outcomes were more comprehensive and accurate, expressing the exact surgical results as it included various related factors rather than single variable. In a previous retrospective report, the success criteria were settled at MIO of ≥ 35 mm and pain score of ≤ 3 with a success rate of 67%, without evaluation of diet or QOL, in addition, their report only investigated the outcomes following the arthroscopic lysis and lavage^26^. The same research group prospectively analyzed the same specific patient variables, revealing that an associated preoperative bilateral masticatory tenderness was the most significant factor affecting the surgical outcomes^27^.

Our results revealed that Wilkes stage III was associated with excellent outcomes based on the multivariate analysis, which was in accordance with the results advocated by previous reports. Furthermore, in a prospective cohort longitudinal study, the outcomes of TMJ arthroscopic disc suturing were correlated to different Wilkes stages, revealing a successful outcome in 69% of the included subjects and that Wilkes II and III stages showed the most successful outcomes^28^. However, these findings were limited by the smaller sample size (32 subjects) and relatively shorter term follow up period (12 months). Likewise, another scholar noticed higher success rates of arthroscopic surgery in the lower than in the advanced Wilkes stages^29^. Alternatively, another study reported that Wilkes stage IV patients were the best candidates for arthroscopy, however the study aimed to compare between joint lavage and operative arthroscopy (without disc suturing) where patient selection with the same Wilkes stage may have alleviated a possible selection bias^30^. Similarly, others reported an equal impact of all Wilkes stages on the surgical outcomes following the arthroscopic surgery^31^.

Furthermore, from our results, the multivariate regression analysis revealed the duration of disease course as a significant prognostic indicator impacting the surgical outcomes. It was reported that the more duration of disease was, the more shortening of the displaced disc and more anteriorly displaced followed by condylar degeneration. The severity of these degenerative changes might restrict alleviating the effects caused by disease and hence the surgical outcomes^32^.

Reviewing the available literature, we found most reports evaluating different treatment modalities for management of TMJ illness using mainly clinical assessment tools, with fewer articles reporting the radiological assessment^8,16^. In the current research, we, for the first time, quantitatively assessed the disc displacement distance preoperatively versus the disc reposition distance postoperatively using MRI. More importantly, we demonstrated the stability of the disc position following surgery and through the longest follow up visit by the difference between the quantitative measurements at immediate and late postoperative follow ups, which is clearly more accurate than simply reporting the disc position with the conventional visual inspection method as reported in all previous articles^8,16^. A single previous study reported the application of the two circle method to quantitatively analyze disc position in MRI, however using angular rather than linear measurements as in our report^19^.

A prognostic nomogram was implemented to predict the prognostic variables most associated with higher chances of successful disc repositioning, reflected by postoperative new bone formation and condylar remodeling as an important index of treatment outcome which can reverse the effects caused by ADD^33^. That nomogram concluded that age of onset, nocturnal bruxism, disc morphology, bone marrow density, Wilkes stages, and postoperative splint therapy were significantly associated with bone remodeling after arthroscopic surgery. From the results of the current study, a preferred surgical outcome was revealed in the condylar height differences between preoperative and 4 years postoperative follow-up interval. This can be explained as follows: firstly; the inclusion of younger group of patients with an active growth potential, secondly; the condyle remodels under pressure by functional capacitation of the repositioned disc which interrupts an abnormal stress from different directions during mandibular movements.

Considering the strengths of this report, the current study presents several advantages including: (1) Preoperative identification of predictor variables is important for helping the treating surgeon to predict the surgical outcomes and to give the patient a fair view of the possible benefits of the suggested surgery, (2) This study acts as a template for case selection algorithm prior to the arthroscopic surgery.

Still, the current research poses some limitations. First, this study was retrospective in design, meaning that weaknesses occur inherent for this study type. During the time period investigated, no formal examination protocol was used and therefore the data should be interpreted with caution. However, the findings may be of clinical value and as such calls for a verifying prospective study, which is currently ongoing. Second, although including a large sample size of participants, however, the male proportion was limited and therefore making the correlation with the gender invalid. Third, the current study did not focus on the comorbidities and risk factors, which can be investigated in a future research.

In conclusion, a preoperative identification of specific patient variables is crucial for defining prognosis and guide surgeons for better patient selection. Age, duration of illness, Wilkes staging, and orthodontic treatment are considered significant prognostic factors that can predict the outcomes following the arthroscopic discopexy for management of TMJ closed lock. In other words, preoperative data of older age, longer disease history, advanced Wilkes staging and previous orthodontic treatment should alert the clinician that a lower chance for success may be at hand.
